# Chronic ethanol exposure impairs alveolar leukocyte infiltration during pneumococcal pneumonia, leading to an increased bacterial burden despite increased CXCL1 and nitric oxide levels

**DOI:** 10.3389/fimmu.2023.1175275

**Published:** 2023-05-19

**Authors:** Flávia Rayssa Braga Martins, Maycon Douglas de Oliveira, Jéssica Amanda Marques Souza, Celso Martins Queiroz-Junior, Francisco Pereira Lobo, Mauro Martins Teixeira, Nathalia Luisa Malacco, Frederico Marianetti Soriani

**Affiliations:** ^1^ Department of Genetics, Ecology and Evolution, Institute of Biological Sciences, Federal University of Minas Gerais, Belo Horizonte, Minas Gerais, Brazil; ^2^ Department of Morphology, Institute of Biological Sciences, Federal University of Minas Gerais, Belo Horizonte, Minas Gerais, Brazil; ^3^ Department of Biochemistry and Immunology, Institute of Biological Sciences, Federal University of Minas Gerais, Belo Horizonte, Minas Gerais, Brazil; ^4^ Department of Microbiology and Immunology, McGill University, Montreal, QC, Canada

**Keywords:** alcohol, nitric oxide, pneumonia, streptococcus pneumoniae, CXCL1

## Abstract

Ethanol abuse is a risk factor for the development of pneumonia caused by *Streptococcus pneumoniae*, a critical pathogen for public health. The aim of this article was to investigate the inflammatory mechanisms involved in pneumococcal pneumonia that may be associated with chronic ethanol exposure. Male C57BL6/J-Unib mice were exposed to 20% (v/v) ethanol for twelve weeks and intranasally infected with 5x10^4^ CFU of *S. pneumoniae.* Twenty-four hours after infection, lungs, bronchoalveolar lavage and blood samples were obtained to assess the consequences of chronic ethanol exposure during infection. Alcohol-fed mice showed increased production of nitric oxide and CXCL1 in alveoli and plasma during pneumococcal pneumonia. Beside this, ethanol-treated mice exhibited a decrease in leukocyte infiltration into the alveoli and reduced frequency of severe lung inflammation, which was associated with an increase in bacterial load. Curiously, no changes were observed in survival after infection. Taken together, these results demonstrate that chronic ethanol exposure alters the inflammatory response during *S. pneumoniae* lung infection in mice with a reduction in the inflammatory infiltrate even in the presence of higher levels of the chemoattractant CXCL1.

## 1 Introduction

Alcohol abuse is one of the risk factors associated with premature death and disability worldwide (1). Digestive disease, injuries, cardiovascular diseases, cancer, and infectious diseases are the major causes of deaths associated with harmful alcohol consumption (1). During the severe acute respiratory syndrome virus (SARS-CoV-2) pandemic, an increased volume and frequency of alcohol consumption were observed within the group of people under isolation, which may have direct consequences for the health of the populations during the pandemic recovery (2, 3).

Effects of alcohol metabolism and its consequences in the immune system have been extensively studied, however differences and similarities between acute and chronic exposure during viral, fungal, and bacterial infections are vastly discussed (4–9). Despite this, literature consensus related to ethanol effects relies on the modulation of the inflammatory response during infection, modifying its normal course (8, 10). Chronic alcohol consumption, as reviewed by Malherbe and Messaoudi, is associated with an increase in the systemic inflammatory response through a direct effect on epigenetic changes in progenitor, circulating and tissue-resident cells, which will have a direct effect during an infection disease (11).

According to the World Health Organization, *S. pneumoniae*, which is included in the group of gram-positive bacteria, is associated with thousands of deaths yearly and one of the risk factors for this infection is alcohol abuse (12–14). Detection of pathogen associated molecular patterns of *S. pneumoniae* by intra- and extracellular pattern recognition receptors on immune cells initiates a proinflammatory response that includes the production of cytokines, chemokines, nitric oxide (NO) and leukocyte recruitment (15, 16). Resident macrophages are essential in this process, coordinating the correct recruitment of neutrophils and the resolution process (17). Neutrophils, in turn, will be the vast majority of migrating cells during pneumococcal pneumonia and their effector functions are essential to limit bacterial spread (18, 19). The C-X-C motif chemokine receptor 2 (CXCR2) ligands, CXCL1 and CXCL2, are critical molecules for neutrophil chemoattraction and host defense against bacterial pneumonia (20, 21).

Pneumococcal infection also induces production of NO mostly by the inducible NO synthase isoform (NOS2), supporting the orchestration of cellular activities during inflammatory response, which includes the production of several cytokines and chemokines such as TNF-α, interleukin 8, and CXCL2 (22–24). In addition to the importance of NO to control pneumococcal viability into the lungs, NOS2 deficient mice are protected during bacteremia caused by *S. pneumoniae*, revealing a contrast effect of NO (25).

Hulse bus and cols., recently demonstrated that moderate intoxication with ethanol increased expression of *Cxcl1* and *Cxcl2*, neutrophil infiltration, and bacterial load into the lungs of mice infected with *S. pneumoniae* infection (5). The aim of this article was to investigate the inflammatory mechanisms involved in pneumococcal pneumonia, such as NO and chemokine production, and cellular infiltration to infectious sites, that may be associated with the chronic ethanol exposure. Our data revealed that following chronic ethanol exposure, mice produced increased amounts of CXCL1 in serum and bronchoalveolar lavage fluid (BALF) after *S. pneumoniae i*nfection along with increased NO levels. Although we observed higher levels of CXCL1 in ethanol infected mice, our results demonstrated decreased neutrophil and macrophage infiltration into the airways of infected alcohol-fed mice. Decreased amounts of leukocytes into the airways were associated with a higher bacterial burden in alcohol-fed mice, however no changes in lethality of infected mice were observed.

## 2 Material and methods

### 2.1 Animals and chronic ethanol exposure

Male C57BL/6J-Unib mice were purchased from the Central Animal Facility of the Federal University of Minas Gerais, Brazil. All experiments received prior approval from the Ethics Committee on the Use of Animals (protocol number: 4/2015) and followed the guidelines of the National Council for the Control of Animal Experimentation (CONCEA, Brazil). Mice were specific pathogen-free and were randomly allocated to polysulfone minisolators, without environmental enrichment and filtered air. Dry food was freely available, light was sustained at 12 hours light/12 hours dark, and temperature was maintained 23 ± 2°C.

Five-week-old mice were initiated to ethanol exposure with a 5% (v/v) ethanol solution in their freely available drinking water during the first week of treatment, 10% (v/v) ethanol in the second week, and 20% (v/v) ethanol solution from the third to the twelfth week (26). Control mice had free access to water during the same period. According to Malacco et al., 2020, the ethanol exposure protocol did not change mice weight gain and generated an average blood alcohol concentration of 200 milligrams per deciliter at the end of twelve weeks exposure. Ethanol treatment was suspended immediately prior infection.

### 2.2 Bacterial culture and infection

Culture of *S. pneumoniae* (ATCC 6303 serotype 3) was performed following protocols established by Tavares et al., 2016 (27). The bacterial inoculum was measured by absorbance until reaching the mid-log phase, centrifuged, and diluted in sterile saline to a concentration of approximately 5x10^4^ CFU in 40 µL. The inoculum was instilled intranasally into mice anaesthetized by inhalation of 3% isoflurane. The inoculum was confirmed by plating serial dilutions of the bacterial suspension on blood agar.

### 2.3 Colony forming unit (CFU)

One day post infection mice were euthanized with ketamine (100 mg/kg) and xylazine (6 mg/Kg) and the lungs were sterile harvested. Bacterial load was analyzed from lung tissue macerated in sterile phosphate-buffered saline (PBS), diluted and plated on blood agar. CFUs were counted after overnight incubation at 37°C, 5% CO_2_ atmosphere.

### 2.4 Histopathological analysis

Left lung lobes were fixed in 4% formalin for 24 hours, progressively dehydrated in alcohol and embedded in paraffin blocks. 5 µm sections were cut from each tissue, placed on slides and stained with hematoxylin and eosin (H&E). Histopathological score analyses of lung slides were performed using the intensity criteria of (i) vascular inflammation (0–4), (ii) airway inflammation (0-4), (iii) parenchymal inflammation (0-5) and (iv) polymorphonuclear cell infiltrate (0-5) (28). The severity of the inflammatory lesions was then classified as absent (0-1), mild (2-5), moderate (6-9), intense (10-13) and severe (14-18). Representative images of the slides were taken under a light microscope at 10x and 40x magnification to better illustrate the phenotypes. Data are expressed as a percentage of animals showing the degree of histopathological lesion described above.

### 2.5 Cellular infiltrate analysis

Blood plasma and bronchoalveolar fluid (BALF) were obtained as previously described (29). Briefly, blood was harvested from inferior cava vein with heparinized Pasteur’s pipette and bronchoalveolar lavage were realized with 2 milliliters of cold PBS, after trachea exposure. BALF was centrifuged 320 *x g* for 10 min at 4°C, and the supernatant was stored at -20°C for chemokine analysis. Total cells in BALF were analyzed by counting in the Neubauer chamber with Turk’s solution. Differential cell counts were obtained by rapid panoptic-stained cytospin preparation (Laborclin) and double-blind observation under light microscopy using cell morphology (nuclear shape and nucleus/cytoplasm ratio) and staining criteria.

Myeloperoxidase (MPO) and N-acetylglucosaminidase (NAG) were used as indirect measures of neutrophil and macrophage accumulation in the lungs, respectively, following the established protocols of Barcelos et al, 2005. Briefly, the collected lungs were homogenized, the contents of the cell granules were released after three rounds of freezing in liquid nitrogen and the supernatant contents were used for the assay. The MPO and NAG standard curves were quantified from a known amount of each cell in a parallel experiment. The resulting colorimetric reaction was read in a spectrophotometer and the results were expressed in relative units per mg of tissue (30, 31).

### 2.6 Blood cells counting

Blood was collected in EDTA tubes. Leukocyte quantification and differentiation was performed using a Celltac MEK-6500K hemocytometer (Nihon Kohden). Plasma was obtained after centrifugation at 600 x g for 10 min at 4°C and stored at -20°C until further investigation.

### 2.7 Nitric Oxide (NO) measurement

NO production in BALF was evaluated indirectly through nitrite formation, by Griess method (32). Briefly, 50 μL of Griess reagent (1% sulfanilamide and 0.1% naphthylethylenediamine in 2.5% phosphoric acid) was added to 100 μL of BALF samples. After 10 min, the absorbance of the samples was measured at 540 nm. Nitrite quantification was done using a nitrite standard curve.

### 2.8 Chemokine measurement

Levels of CXCL1 in plasma and BALF and IL-6, TNF-α and CCL2 in BALF were obtained using DuoSet Enzyme-Linked Immunosorbent Assay kits (R&D 348 Systems). The assays followed the manufacturer’s instructions. The absorbance found in the samples was compared to the specific standard curve of known values for each cytokine/chemokine evaluated.

### 2.9 Statistical analysis

Experiments were done at least twice and randomically and double-blind analyzed. Graphs and statistical analysis were performed using Graph Pad Prism 8. Differences between different groups were analyzed by Two-way analysis of variance (ANOVA) and Tukey’s *post hoc* test. Statistical significance was considered when p<0.05. Survival analysis used the Log Rank test.

## 3 Results

### 3.1 Chronic ethanol exposure is related to increased bacterial load into the lungs

**Figure 1:**
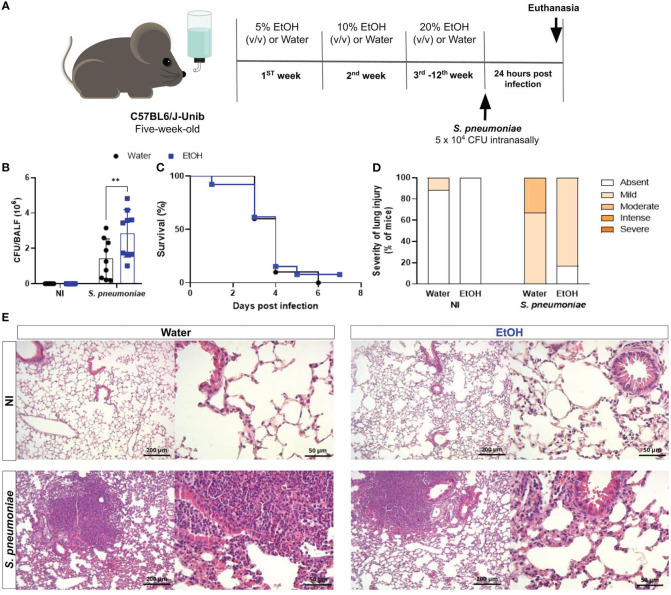
Chronic alcohol exposure impaired bacterial clearance with no effects on survival rate after *S. pneumoniae* infection. **(A)** Methodological scheme of exposure to ethanol and infection. Five-week-old male C57BL6/J-Unib mice started the chronically exposure to ethanol with a 5% (v/v) alcoholic solution. Alcohol concentration doubled after one week until the third week and remained stable (20% v/v) at the end of treatment. After alcohol treatment, mice were infected with 5 x 10^4^ CFU of *S. pneumoniae*. After 24h hours of infection, mice were euthanized. **(B)** Bacterial Load into the lungs after infection. **(C)** Lethality curves. **(D)** Percentage of mice with different severity of lung inflammatory lesions and **(E)** representative H&E stained lung section images under light microscopy at 10x and 40x magnification. Data are presented as the mean ± SD (6 to 9 mice per group). ** Significantly different (p < 0.01) by two-way ANOVA analysis test.

Proper bacterial clearance is critical for the improvement in the progression of infectious diseases (19). To assess the impact of chronic ethanol exposure on bacterial clearance, at the end of twelve weeks treatment, mice were intranasally infected with *S. pneumoniae* and twenty-four hours later were euthanized, lungs and plasma were harvested (**Figure 1A**). Results demonstrate recovery of bacteria from both infected groups, demonstrating an established infection. Nevertheless, alcohol-fed mice had a higher number of viable bacteria into the lungs compared to untreated mice (**Figure 1B**). We hypothesized that these animals would be more susceptible to infection, since bacterial clearance was greatly damped in alcohol-fed mice. We evaluated mice survival after *S. pneumoniae* infection and, curiously, we did not observe significant differences between groups (**Figure 1C**). These results encouraged us to investigate the inflammatory mechanisms that might be involved in mice survival even with increased bacterial load.

Histopathological analyses of the inflammatory process into the lungs were observed and uninfected animals in the ethanol-exposed and unexposed groups showed little or no severity of lung injury (**Figures 1D, E**). In contrast, after twenty-four hours of infection with *S. pneumoniae*, we observed an increase in the frequency of mice with increased severity of inflammatory lesions in the lung tissue (**Figure 1D**). The frequency of severe inflammatory lesions was higher in the infected group not exposed to ethanol (**Figure 1D**). Images of representative tissue sections show a more prominent inflammatory infiltrate into the lungs of water-treated infected mice compared to ethanol-exposed infected mice (**Figure 1E**).

### 3.2 Increased NO production is associated with chronic ethanol exposure

**Figure 2:**
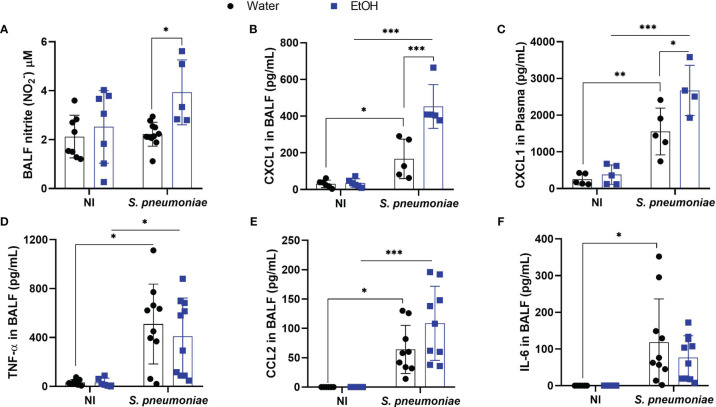
Alcohol-fed mice had higher production of NO, CXCL1 in BALF and plasma 24 hours after pneumococcal infection. After alcohol treatment, mice were infected with *S. pneumonia*e and 24h hours post infection mice were euthanized, BALF and blood plasma was collected to determine **(A)** NO production through Griess reaction and for analysis of pró-inflammatory mediators. CXCR2 ligand, chemokine **(B)** CXCL1 on BALF supernatant and **(C)** plasma, **(D)** TNF-α in BALF, **(E)** CCL2 in BALF and **(F)** IL-6 in BALF. Data are presented as the mean ± SD (4 to 9 mice per group). *Significantly different (p < 0.05), **Significantly different (p < 0.01) by two-way ANOVA analysis test. ***Significantly different (p < 0.001) by two-way ANOVA analysis test.

Chronic ethanol consumption is associated with increased NO production in liver, kidney, and lung (33–35). Although cellular NO signaling has an ambiguous role during pneumococcal infection, we investigated whether chronic exposure would alter this inflammatory mechanism (25). To understand NO production in the alveoli of ethanol-exposed mice, we assessed NO production through nitrite formation. Twenty-four hours post-infection, alcohol-fed mice produced increased amounts of NO in BALF, whereas untreated infected mice showed a similar NO production to uninfected mice (**Figure 2A**). As NO signaling has been linked to production of other inflammatory mediators, we investigated whether this increase in BALF NO levels would also be associated with an increase in CXCL1 (16, 23, 24, 36).

### 3.3 Chronic alcohol exposure increases CXCL1 released in BALF and blood in pneumococcal infection

The orchestration of the inflammatory response is essential for the removal of bacteria from the infectious site (19). This process ranges from pathogen recognition to the recruitment and activation of phagocytes (37). CXCL1 is an important chemokine responsible for the influx of neutrophils into the lungs during Gram-positive bacterial pneumonia (20). Twenty-four hours after infection, alcohol-fed mice produced significantly higher amounts of CXCL1 in BALF compared to infected untreated mice (**Figure 2B**). At the same time point, the release of CXCL1 in plasma increased in both infected mice, with alcohol-fed mice showing higher levels of this chemokine (**Figure 2C**). Although CXCL2 is also a CXCR2 ligand and plays an important role in neutrophil recruitment to sites of infection, CXCL2 levels did not change in the blood or alveoli of both infected or uninfected groups. (**Supplementary Figure 1**). We also assessed BALF levels of other important pro-inflammatory mediators during pneumococcal pneumonia such as TNF-α, IL-6 and CCL2. The levels of these mediators increased in the alveoli twenty-four hours after infection, but there were no statistical differences between ethanol-treated infected mice and water-treated infected mice groups (**Figures 2D–F**).

### 3.4 Decreased neutrophil and macrophage migration to alveoli is associated with ethanol exposure also in the presence of high levels of chemokines

**Figure 3:**
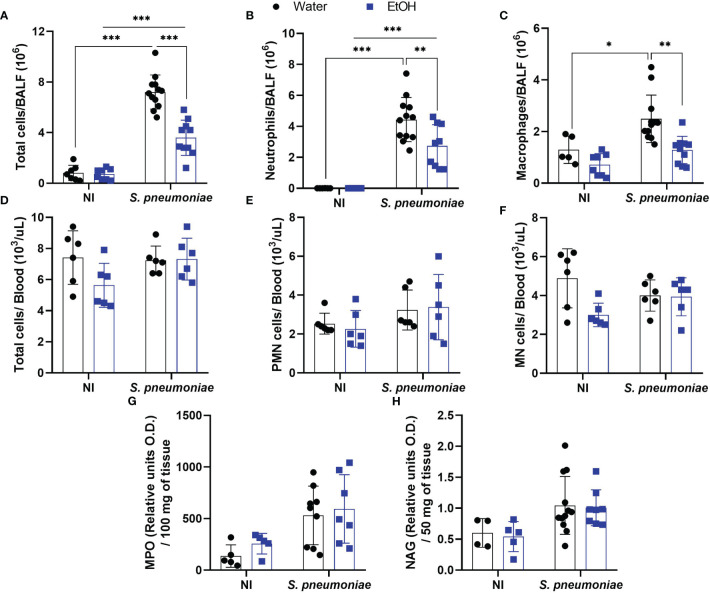
Alcohol-fed mice have decreased neutrophil and macrophage infiltration into airways 24 hours post pneumococcal infection. After alcohol treatment, mice were infected with *S. pneumonia*e. After 24h hours of infection, mice were euthanized, BALF, blood and lungs were collected to determine the infiltration of inflammatory cells. **(A)** Total cells, **(B)** Neutrophils and **(C)** Macrophages count in BALF. **(D)** Total cells, **(E)** polymorphonuclear cells and **(F)** mononuclear cells into the blood. **(G)** Neutrophils and **(H)** macrophages infiltrated into the lung parenchyma. Data are presented as the mean ± SD (7 to 11 mice per group). * Significantly different (p < 0.05), **Significantly different (p < 0.01), ***Significantly different (p < 0.001) by two-way ANOVA analysis test.

To understand the significantly increased bacterial load into alcohol-fed mice even in the presence of higher levels of NO and CXCL1, we decided to investigate inflammatory cell influx into the infection site. Ethanol consumption did not affect the total cell amount in alveoli before the bacterial challenge (**Figure 3A**). After infection, alcohol-fed mice demonstrated a significantly decreased number of cells into the airways compared to non-treated infected mice (**Figure 3A**). To analyze the immune cell profile that migrates to alveoli during *S. pneumoniae* infection, we performed a double-blind differential cell count. Both groups of infected mice showed a massive neutrophil and macrophage infiltration, even though alcohol-fed mice presented a decreased migration of both cell types into alveoli (**Figures 3B, C**).

Despite the reduced migration of leukocytes into the BALF of infected mice exposed to ethanol, the total amount of circulating leukocytes in the blood was not altered in any of the experimental groups (**Figure 3D**). When polymorphonuclear and mononuclear cells in the blood were assessed, no differences were observed between any of the groups (**Figures 3E, F**). At the same time post-infection, we also observed no difference in the indirect quantification of neutrophils or macrophages in the lung parenchyma by the NAG and MPO assays (**Figures 3G, H**).

## 4 Discussion

**Figure 4:**
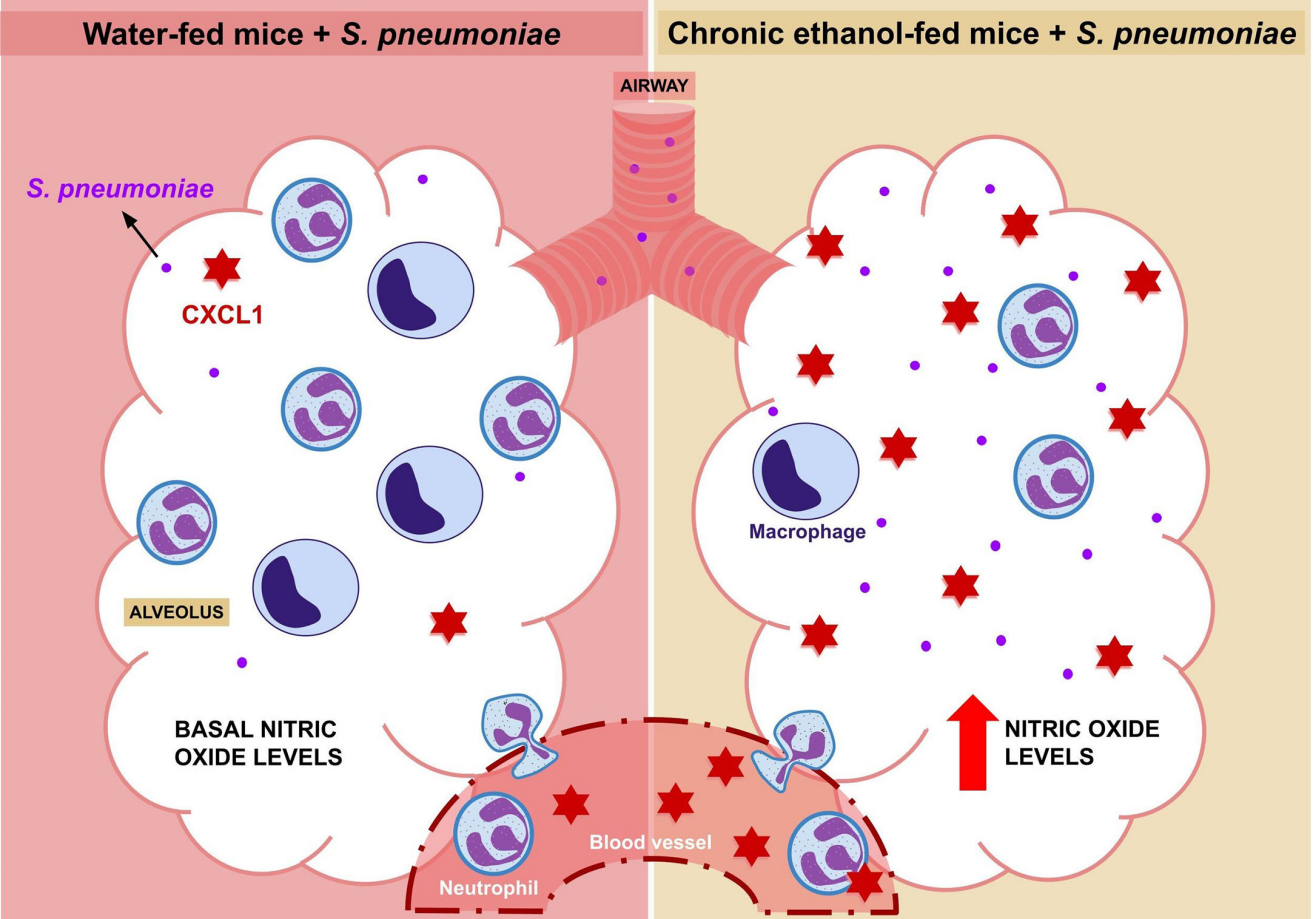
Elucidative Design. After 24 hours of infection with Streptococcus pneumoniae, an increase in the migration of macrophages and neutrophils into the alveolus is observed. However, this migration is reduced in alcohol-fed mice, although this group also shows an increase in CXCL1 in plasma and BALF. In contrast to water-fed mice, which have low levels of nitric oxide at baseline, alcohol-fed mice have a higher bacterial load and higher alveolar nitric oxide production compared to untreated mice.

Alcohol exposure is already known as a risk factor for *S. pneumoniae* infection by affecting the immune response during pneumococcal pneumonia, although the mechanisms involved with chronic alcohol consumption are not yet fully understood (9, 38–40). Here we demonstrated that increased bacterial load in alcohol-fed mice is associated with decreased leukocyte migration into the alveoli, reduced frequency of severe lung inflammation and systemic and local CXCL1 release during infection (**Figure 4**).

Neutrophil migration into the lungs is a critical mechanism during pneumococcal infection. Neutrophil depletion reduced clearance of *S. pneumoniae* in a mouse model of infection (41). In addition, antimicrobial mechanisms released by activated neutrophils are critical for controlling bacterial growth in the lung, as serine protease-deficient animals have impaired antimicrobial defense and increased mortality after infection (37, 42). Here, we associate the reduced number of alveolar neutrophils with the higher bacterial load in chronically alcohol-fed mice. We observed that the decreased leukocyte amounts in the alveoli of chronically ethanol-exposed and infected mice was reflected as a decrease in the frequency of inflammatory lesions in the lungs. These data show that even when the bacterial load was increased in animals chronically exposed to ethanol, the severity of the lung lesions, observed 24 hours after infection with *S. pneumoniae*, was smaller due to the reduced migration of leukocytes to the site of infection.

Curiously, this phenotype did not affect mortality. This could be due to the balance between an excessive inflammatory response and microbial burden control, or it could be related to higher mortality rate found in this inoculum (43). The ambiguous role of neutrophils in microbial inflammation is also still under investigation. Studies have already shown that attenuation of neutrophil migration is associated with reduced lung injury by preventing the damage caused by neutrophil activation and migration (44). However, the mechanisms related to the survival of alcohol-fed animals with fewer neutrophils and higher fungal load need to be further investigated. Furthermore, one of the limitations of this article is the observation window of leukocyte migration, which is limited to the first 24 hours of infection.

Recently, Hulsebus et al. (2022) demonstrated that moderate alcohol consumption increased the expression of *Cxcl1* in mice lung tissue during pneumococcal infection and was accompanied by the increase in neutrophil infiltrate (5). However, previous results from our research group have shown that although chronic ethanol exposure induces elevated levels of CXCL1 in serum and BALF during *Aspergillus fumigatus* infection, it is also associated with a decrease in neutrophil infiltration, due to a decrease in CXCR2 expression in these cells (7). Moreover, we also demonstrated an impairment in neutrophil effector functions and a higher susceptibility to fungal infection (7). Without altering mice susceptibility to pneumococcal pneumonia, our data together suggest that chronic ethanol exposure induces important inflammatory changes during acute pneumonia, impairing adequate neutrophil migration to the site of infection. This phenotype is also observed in sepsis, a decreased neutrophil migration which is associated with increased NO release and consequent overproduction of pro-inflammatory mediators, resulting in failure of neutrophil response and lethality (45, 46).

Acute exposure to ethanol decreases granulocyte migration to the lungs during pneumococcal infection that is related to a decrease in granulopoiesis in the bone marrow (BM) of ethanol-exposed mice (47). Recently, we demonstrated that the same regimen of ethanol exposure used here does neither change the number of circulating cells in treated animals, nor the number of granulocyte precursors in BM (7). Here, we did not observe any change in the number or cell type of circulating leukocytes that could be altered by chronic exposure to ethanol. These data do not exclude the possibility that these cells have altered functionality, but highlight the fact that the decrease in cell migration to the alveoli occurs independently of the number of circulating cells. After our analyses also revealed an equal number of neutrophils and macrophages in the lung parenchyma, we can assume that the chronic effect of ethanol observed is related to the lack of migration of these cells directly to the alveoli.

Ethanol exposure increases NO released from endothelial cells and is associated with liver injury in alcoholic hepatitis (33, 48, 49). NO is known to have a role in controlling cytokine and chemokine expression (50), which could be a promising target for inflammatory diseases. This important biological mediator alters the expression pattern of genes by disrupting the activity of enzymes responsible for post-transcriptional modifications of histones, such as histone acetyltransferases and methyltransferases (51). The modification of the epigenetic code and the interaction of the transcriptional machinery with gene promoters is a determining factor for the inflammatory cell profile (52). In conjunction with the increased production of NO in the BALF of ethanol-exposed mice during pneumococcal infection, we observed a significant increase in the amount of CXCL1 in the blood and BALF. This increased production of chemokine may be due to nitric oxide and its role in epigenetic modulation during the inflammatory response, but this relationship needs to be further investigated during chronic ethanol treatment. Indeed, as highlighted by Malherbe and Messaoudi, the systemic effects of ethanol metabolism and its by-products in regulating gene expression through epigenetic modifications will be extremely important during infection and homeostasis (11).

The development of invasive pneumococcal disease is more common in people with a history of alcohol abuse, about 21% compared with 6% in the general population (53). Therefore, it is important to search and understand the pathophysiology of pneumococcal infection in patients with a history of alcohol abuse, to improve therapeutic interference and preventive measures for those affected. Health surveillance is also important to ensure that alcoholics receive adequate medical care and have a better outcome during pneumococcal pneumonia.

In conclusion, we demonstrate that chronic ethanol exposure in mice is associated with increased bacterial load and decreased leukocyte migration during pneumococcal pneumonia, even in the presence of higher levels of CXCL1. This profile may be also associated with increased BALF NO production, highlighting the ambiguous role of NO during pneumococcal infection.

## Data availability statement

The datasets presented in this study can be found in online repositories. The names of the repository/repositories and accession number(s) can be found in the article/**Supplementary Material**.

## Ethics statement

The animal study was reviewed and approved by Ethics Committee on the Use of Animals at Federal University of Minas Gerais - CEUA UFMG.

## Author contributions

FS and NM guided the development of the project and the writing of the manuscript. FM, NM and JS collected the samples and performed the experiments. MO performed the bioinformatics analysis and participated in statistical analysis. FM analyzed the data results and statistical analysis, illustrated the results and wrote the manuscript. CQ-J performed the histopathological analyses. FL and MT participated intellectually in the development of the project. All authors contributed to the article and approved the submitted version.
