# Supplementary material for: Chronic ethanol exposure impairs alveolar leukocyte infiltration during pneumococcal pneumonia, leading to an increased bacterial burden despite increased CXCL1 and nitric oxide levels
Source: Front Immunol. 2023 May 19;14:1175275. doi: 10.3389/fimmu.2023.1175275 (PMC10235596; doi:10.3389/fimmu.2023.1175275)
Supplement: Supplementary file 1 [file Image_1.pdf]

Supplementary Figure 1

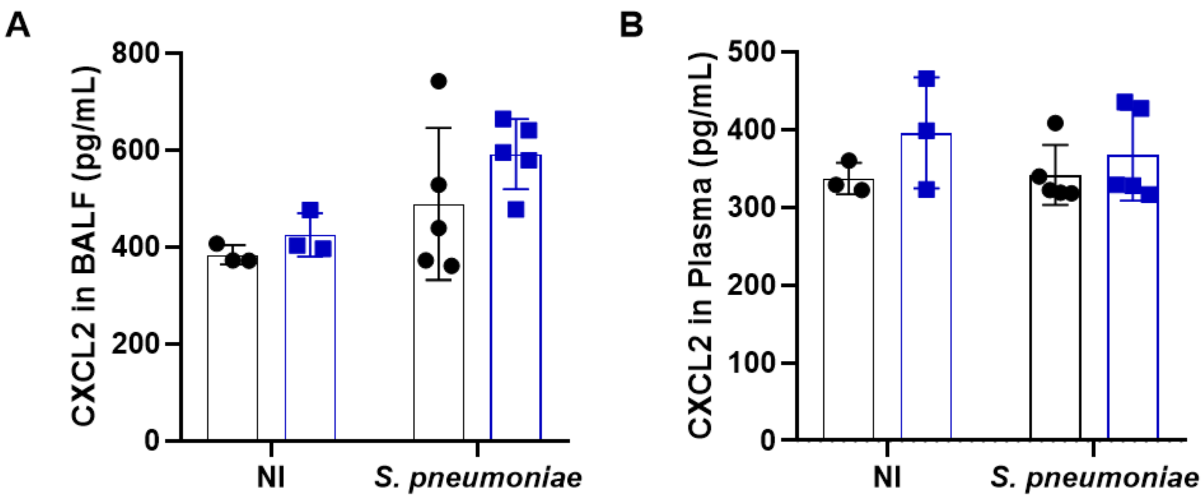

Supplementary Figure 1. CXL2 levels in BALF and plasma did not change after twenty-four hours of pneumococcal pneumonia. Male C57BL/6J Unib mice were obtained from the Central Animal Facility of the Federal University of Minas Gerais, Brazil. All experiments were approved in advance by the Ethics Committee for the Use of Animals (protocol number: 4/2015). Five-week-old mice were initiated to ethanol exposure with a 5% (v/v) ethanol solution in their freely available drinking water during the first week of treatment, 10% (v/v) ethanol during the second week, and 20% (v/v) ethanol solution from the third to the twelfth week. At the end of the ethanol exposure, $5x10^4$ CFU of *S. pneumoniae* were instilled intranasally into mice anaesthetised by inhalation of 3% isoflurane. Twenty-four hours after infection, mice were euthanised and blood and bronchoalveolar lavage fluid were collected. CXCL2 levels in (A) BALF and (B) plasma were determined using DuoSet Enzyme-Linked Immunosorbent Assay kits (R&D 348 Systems). Assays were performed according to the manufacturer's instructions. Data are presented as the mean ± SD (3 to 8 mice per group). Analysed by two-way ANOVA.
